# Measuring levels of person-centeredness in acute care of older people with cognitive impairment: evaluation of the POPAC scale

**DOI:** 10.1186/1472-6963-13-327

**Published:** 2013-08-19

**Authors:** Anita Nilsson, Marie Lindkvist, Birgit H Rasmussen, David Edvardsson

**Affiliations:** 1 Department of Nursing, Umeå University, Umeå, Sweden; 2 Department of Statistics, Umeå University, Umeå, Sweden; 3 School of Nursing and Midwifery, La Trobe University, Melbourne, Australia

**Keywords:** Person-centred care, Nursing, Older people, Cognitive impairment, Scale, Measurement, Intervention

## Abstract

**Background:**

Person-centeredness is increasingly advocated in the literature as a gold-standard, best practice concept in health services for older people. This concept describes care that incorporates individual and multidimensional needs, personal biography, subjectivity and interpersonal relationships. However, acute in-patient hospital services have a long-standing biomedical tradition that may contrast with person-centred care. Since few tools exist that enable measurements of the extent to which acute in-patient hospital services are perceived as being person-centred, this study aimed to translate the English version of the Person-centred care of older people with cognitive impairment in acute care scale (POPAC) to Swedish, and evaluate its psychometric properties in a sample of acute hospital staff.

**Methods:**

The 15-item POPAC was translated, back-translated and culturally adjusted, and distributed to a cross-sectional sample of Swedish acute care staff (n = 293). Item performance was evaluated through assessment of item means, internal consistency by Cronbach’s alpha on total and on subscale levels; temporal stability was assessed through Pearson’s product correlation and intra-class correlation between test and retest scores. Confirmatory factor analysis was used to explore model fit.

**Results:**

The results indicate that the Swedish version POPAC provides a tentatively construct-valid and reliable contribution to measuring the extent to which acute in-patient hospital services have processes and procedures that can facilitate person-centred care of older patients with cognitive impairment. However, some questions remain regarding the dimensionality of POPAC.

**Conclusions:**

POPAC provides a valuable contribution to the quest of improving acute care for older patients with cognitive impairment by enabling measures and subsequent accumulation of internationally comparable data for research and practice development purposes. POPAC can be used to highlight strengths and areas for improvements in care practice for older patients, and to illuminate aspects that risk being overlooked in busy acute hospital settings.

## Background

It has been argued that a more person-centred pathway in acute hospital services, where bio-psychosocial needs are in focus for care of older patients with cognitive impairment, improve outcomes for patients, family members and staff [1,2]. However, acute hospital services commonly have a strong medical focus built on medical routines and organisational efficiency; something which may contrast with the person-centred care (PCC) philosophy that aims to incorporate psychosocial needs as much as medical needs especially in relation to older patients and the specific needs that accompany a cognitive impairment [1,3,4]. The limited holistic perspective that is implied in disease-centred, not person-centred, care for older patients and their needs has been linked to several complications during hospitalisations such as pressure sores, incontinence, falls [5], malnutrition [6,7], functional decline [8], delirium [9,10], longer hospital stays and increased mortality [11].

The contemporary business model of health service provision prioritises attending to medical needs and discharging patients as soon as their medical condition is stable, which risks giving patients’ biopsychosocial needs lower priority, which may result in dissatisfaction with care [1,4,12]. It has been shown that disease-oriented and efficiency-driven ward structures work against the provision of PCC [13]. Barriers to PCC provision can also be found in having a busy and strongly medicalised environment and in a lack of staff consensus about models of care for older patients with cognitive impairment [1]. Acute hospital services that contain such barriers risk failing to meet the biopsychosocial needs of older patients [1], and risk missing out on economic and personal benefits such as lower costs and higher staff and patient satisfaction that have been linked to increasing hospital person-centeredness [14-17].

To increase the quality and safety of acute hospital-based health services to the ageing population, there is a need to explore further and compare the extent to which acute hospital services provided to older patients are perceived as being person-centred in relation to the needs that accompany ageing and cognitive decline [18-20]. To enable such explorations and comparisons, we need valid, reliable tools that provide comparable data on levels of perceived person-centeredness in various services. To date, however, tools that enable valid and reliable measurements for national and international comparisons are lacking. Developing measurements further is required, as person-centeredness is considered to be an indicator of contemporary high quality health services of older people with cognitive impairment [11,19].

Reporting valid and reliable tools for this purpose makes possible the accumulation of internationally comparable data on the extent to which acute hospital health services are person-centred in relation to older patients with cognitive impairment. A recent review identified 12 eligible tools for measuring PCC internationally, where only three were adequate for use in acute hospital settings [20]. However, none of the three tools were developed to measure PCC for older patients with cognitive impairment in the acute hospital environment.

To fill that gap in knowledge, the Person-Centred care of Older People with Cognitive impairment in Acute Care (POPAC) scale was developed to enable quantitative exploration of perceived levels of person-centeredness in acute settings and highlight areas in need of improvement. The scale was developed in an Australian version and tested in a sample of nursing staff at a metropolitan acute hospital in Melbourne, Australia, and was found valid and reliable [18]. Further testing of the validity, reliability and applicability of the scale in other samples and settings was recommended, and permission was granted to use and test the POPAC in a Swedish acute care context. Thus, the aim of the present study was to translate POPAC to Swedish and evaluate its psychometric properties in a sample of acute hospital staff members in Sweden.

## Methods

A quantitative descriptive survey was conducted.

### Instrumentation

The POPAC scale contains 15 items, formulated as statements relating to the extent to which different care procedures are perceived as being person-centred. The scale aims to measure staff perceptions of PCC, and responses are given on a six-point Likert-type scale ranging from (1) ‘never’, (2) ‘very rarely’, (3) ‘rarely’, (4) ‘frequently’, (5) ‘very frequently’, to (6) ‘always’. The scale consists of three subscales; Subscale I ‘using cognitive assessments and care interventions’ consists of items 1–5, subscale II ‘using evidence and cognitive expertise’ of items 6–8, and subscale III ‘individualising care’ of items 9–15, (see Table 1 for included items). Scale scores are to be summed on total and sub-scale levels, and total scores can range between 15 and 90 with higher scores indicating a higher degree of person-centeredness. The original Australian version POPAC was reported to have satisfactory psychometric properties in the original study, with a total Cronbach’s alpha of 0.87 and subscales Cronbach’s alpha values of 0.74, 0.79, and 0.78. The three subscales explained 53% of the total variance in the original factor model. The content and construct validity of the original POPAC was reported as satisfactory in the original study through expert panel assessments and a stable three factor dimensionality explaining 53% of the variance in original data [18].

**Table 1:** Item performance of the Swedish POPAC scale

**Item**	Mean*	**SD**	**Corrected item-total correlation**	**Cronbach’s alpha if item deleted**
**At my unit,**				
1. We assess the cognitive status of our older patients on admission.	4.54	1.23	.30	.83
2. We make environmental adjustments to avoid over-stimulation in older people with cognitive impairment (e.g. single rooms, noise reductions etc.).	3.27	1.12	.50	.81
3. We diagnose symptoms of cognitive impairment (e.g. dementias, delirium etc.).	3.59	1.13	.32	.82
4. We spend more time with older patients with cognitive impairments as compared to cognitively intact patients.	3.62	0.96	.37	.82
5. We leave older people with cognitive impairments alone in the ward.	3.21	0.94	.38	.82
6. We use evidence-based tools to assess cognitive status of older patients (e.g. the MMSE, SPMSQ, CAM etc.).	2.28	1.32	.36	.82
7. We consult specialist expertise (e.g. psychologist, gerontologist) if we find that a patient has cognitive impairment.	2.96	1.10	.32	.82
8. We use evidence-based care guidelines in the care of older cognitively impaired patients.	2.26	1.09	.52	.81
9. We use biographical information about older patients’ (e.g. habits, interests and wishes etc.) to plan their care.	2.74	1.08	.62	.80
10. We involve family members in the care of older patients with cognitive impairment.	3.66	1.11	.54	.81
11. We provide staff continuity for older patients with cognitive impairments (e.g. the same nurses providing care to these patients as often as possible).	2.65	1.19	.52	.81
12. We systematically evaluate whether or not older patients with cognitive impairment receive care that meets their needs.	2.77	1.18	.55	.81
13. We involve older patients with cognitive impairment in decisions about their care (e.g. examinations, treatments etc.).	3.55	1.18	.47	.81
14. We ensure that older patients with cognitive impairment have tests/ examinations/ consultations in the unit rather than having to go to another department.	3.15	1.18	.45	.82
15. We discuss ways to meet the complex care needs of people with cognitive impairment.	3.18	1.04	.48	.81

*POPAC scale ranging from (1) ‘never’, (2) ‘very rarely’, (3) ‘rarely’, (4) ‘frequently’, (5) ‘very frequently’, to (6) ‘always’.

### Translation

The English version scale was translated to Swedish and back-translated to English by two independent accredited translators and the correspondence between the two versions was deemed to be sufficiently high. A native-speaking Swedish clinical group of five registered nurses, four enrolled nurses and one physician (n = 10) evaluated comprehensibility and cultural suitability of the Swedish version. Items 5 and 14 were identified as being somewhat difficult; these items were marginally adjusted to increase clarity. Item 5 was formulated as 'being without supervision', and item 14 was formulate as 'striving for' in the translated Swedish version [21].

### Sampling and data collection

The sampling strategy was a combination of convenience and total sampling. The participating hospital was selected by convenience, and the staff sampling targeted the total population of staff within the medically oriented clinics within the hospital. Data was subsequently collected from a sample of acute hospital staff at five medically oriented inpatient clinics at a university hospital in Sweden. The inpatient clinics consisted of 14 wards with specialisation in cardiology, infectious diseases, general medicine, neurology, and oncology. The inclusion criteria were being a current staff member involved in patient-related work (assistant nurses, registered nurses, and physicians), and consenting to participation. Current staff records at the time of the study indicated a total eligible sample of N = 578. Staff were informed about the study orally and/or in writing at ward meetings, and were asked to participate by anonymously returning a completed survey by pre-paid response mail. Questionnaires were distributed through staff pigeonholes and by the clinical heads of participating departments. Data were collected between February and April 2012.

### Statistical analyses

Item performance was evaluated through assessment of item means and standard deviations (SD). Construct validity was evaluated by confirmatory factor analysis based on the original three-factor solution. Model fit evaluation involved X^2^/df (normed X^2^), the comparative fit index (CFI), the standardised root mean square residual (SRMR), and the root mean square error of approximation (RMSEA). Cut-offs for acceptable values were set to an X^2^/df of < 3, a CFI of >0.90, a SRMR of < 0.10, and a RMSEA of <0.08 [22]. Internal consistency reliability was evaluated by Cronbach’s alpha, on total and on subscale levels, by corrected item-total correlations, and by inter-item correlation. Cut-offs for acceptable internal consistency were set to a Cronbach’s alpha of >0.7 [23], item-total correlations of >0.3 [24], and inter-item correlations between 0.2–0.4 [25]. Temporal stability was evaluated through correlation between test and retest scores. A sub-sample of 25 staff members were included in the test-retest analysis by completing the scale at two time points three weeks apart. Cut-offs for acceptable temporal stability were set to >0.5 for Pearson’s product moment correlation as well as for intra-class correlation coefficients between test and retest scores. When an individual had fewer than 10% missing items, the missing item value was replaced with the individual mean [26]. Items with negative wording (item 5) were reversed before analysis. The PASW statistics version 20 (SPSS Inc., Chicago, IL, USA) and IBM SPSS AMOS 20 [27] was used to analyse the data.

### Ethics

Ethics approval was obtained from the Regional Ethical Review Board in Umeå, Sweden (Reg. No. 2012–302–32 M). The study complied with the Helsinki Declaration [28].

## Results

From the eligible sample of staff members (N = 578), a total of 293 questionnaires were returned, providing a 51% response rate. As shown in Table 2, most respondents were female (73%), had a mean age of 38.7 (SD 11.23) years, consisted of physicians (22%), enrolled nurses (29%), and registered nurses (49%), and they had worked about nine years on average at the current ward.

**Table 2:** Sample characteristics

	**n (%)**
**Gender** (n = 288)	
Women	212 (73)
Men	79 (27)
**Profession** (n = 291)	
Enrolled nurses	84 (29)
Registered nurses	143 (49)
Physicians	64 (22)
**Mean yrs. (SD)**	
**Age** (n = 289)	38.7 (11.23)
**Health care experience** (n = 288)	15.34 (10.79)
**Experience at current ward** (n =284)	8.97 (8.09)

### Item performance

The mean total POPAC score was 47.45 (SD 9.11) with a skewness of 0.69, and mean values for single items ranged 2.26–4.54 (Table 1).

### Construct validity

**Figure 1:**
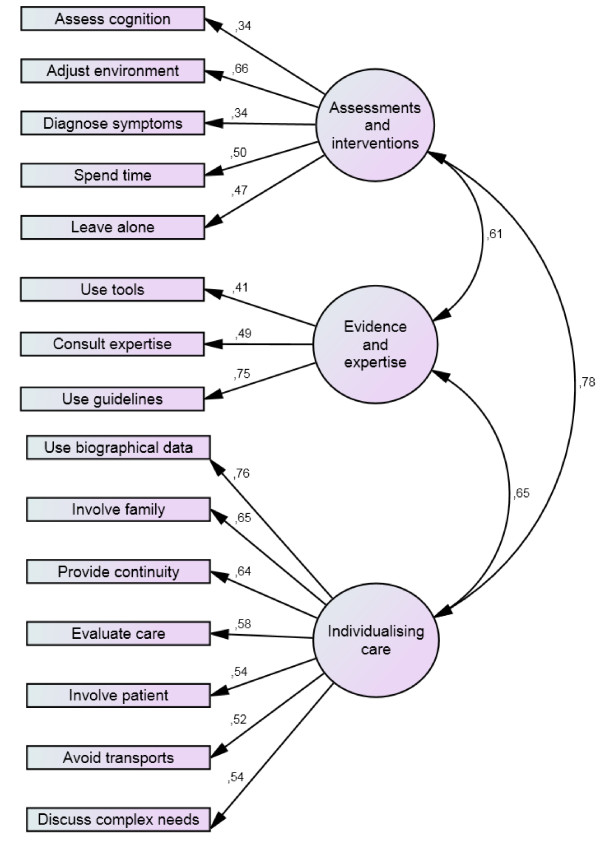
Confirmatory factor analysis of the proposed factors.

The original three-factor dimensionality had acceptable model fit as evidenced through X^2^/df of 2.21, an SRMR of 0.06, and an RMSEA of 0.07 (CI 90% 0.054–0.080). However, the CFI of 0.88 did not quite reach the pre-set cut-off for acceptable model fit. Figure 1 illustrates the model fit through significant correlations between the subscales, and through significant standardised regression weights between indicators (items) and latent constructs (subscales).

### Reliability

Internal consistency reliability was satisfactory for the Swedish version POPAC on total scale level, as evidenced by a Cronbach’s alpha of 0.83, and on subscale level for subscale III (Cronbach’s alpha of 0.80). However, subscales I and II had Cronbach’s alpha values of 0.56 and failed to reach the cut-off of >0.7. All item-total correlations were acceptable as evidenced by values between 0.30–0.62 and the inter-item correlations were satisfactory for all subscales, as evidenced through values of 0.21 (CI 0.03–0.32) for subscale I, 0.31 (CI 0.26–0.38) for subscale II, and 0.37 (CI 0.27–0.56) for subscale III.

### Temporal stability

As shown in Table 3, the temporal stability of POPAC was satisfactory, as supported by correlation coefficients ranging between 0.58 and 0.75 on total and on subscale levels between test and retest.

**Table 3:** Temporal stability for the Swedish version of POPAC (n = 25)

**Scale dimension**	Test mean (SD)*	Retest mean (SD)*	**Pearson correlation**	**Intra-class correlation**
**Total POPAC score**	3.52 (0.57)	3.21 (0.65)	.70*	.70*^a^
**Subscale 1**	4.22 (0.40)	3.88 (0.57)	.63*	.59*^a^
‘*Assessments and interventions*’				
***Subscale 2***	2.77 (0.91)	2.52 (1.04)	.58*	.58*^a^
‘*Evidence and expertise*’				
***Subscale 3***	3.34 (0.74)	3.02 (0.77)	.75*	.75*^a^
‘*Individualising care*’				

*Correlations is significant at a level of 0.01 (two-tailed).

^a^ single measure.

SD = Standard deviation.

## Discussion

This study aimed to translate POPAC to Swedish and evaluate its psychometric properties in a sample of acute hospital staff. The Australian version of the POPAC scale was found reliable and valid through estimates of content and construct validity [18]. The overall result of this study indicates that the Swedish version POPAC is tentatively construct-valid and reliable as a measure of the extent to which acute in-patient hospital wards are perceived to have processes and procedures that facilitate PCC of older patients with cognitive impairment. However, some questions remain regarding the dimensionality of POPAC, as one of the model fit estimates did not reach the cut-off, and as two subscales did not quite reach the reliability targets. Thus, further data is needed on the dimensionality of the tool in other settings and samples, and on the reliability of the subscales when evaluated in other or similar contexts. However, POPAC can make a valuable contribution to the literature by being the first measurement tool that can provide internationally comparable data on the extent to which acute in-patient wards are perceived to be person-centred, and thus contribute to exploring, comparing and improving the care of and services to older patients with cognitive impairment in acute care settings.

Previous studies have shown that up to 63% of cognitive impairment among older patients (>75 years) in general hospitals pass by unrecognised [29]. This makes the provision of PCC very difficult as PCC builds on an awareness of individual needs and abilities [30]. It has also been shown that the presence of individualised care plans for in-patients with dementia were limited to 15% of patients in acute hospital wards in 1999 [31]; a disconcerting finding which remains in some places thirteen years later [32]. Some of the POPAC items can highlight the extent to which staff perceive patient cognitive status being assessed, if evidence-based tools are used for this purpose, and also if evidence-based guidelines are used for providing care for this frail patient group. Thus, using the tool in practice can highlight if cognitive status is perceived as being assessed appropriately with reliable and valid tools, and if staff incorporate guidelines to facilitate high quality care for this population. It is essential to incorporate evidence-based guidelines for the care of older people with cognitive impairment in acute hospital settings, as staff members in general may not have the geriatric skills and expertise to provide contemporary high quality care that meets the needs of this population. Guidelines offer support and structure to providing evidence-based care to older patients who are not always able to communicate their needs.

The study findings indicated that the POPAC ratings differed somewhat between the Swedish and Australian samples, with higher average item ratings within the Australian sample. The mean total scores were 3.77 (SD 0.65) in the Australian sample [18] compared with the Swedish mean total score of 3.16 (SD 1.12). This would indicate that the extent to which care processes and procedures are perceived to be person-centred in relation to older people with cognitive impairment are higher in the Australian study [18]; a preliminary finding that deserves further study. If these differences can be explained by a higher prevalence or a more frequent use of guidelines in the Australian clinical setting, or by different training or regulations between the countries, remains unknown and needs further study. The Swedish sample included medical physicians whereas the original Australian sample did not. This means the cross-sample comparisons need to be interpreted with caution, as the two samples are not entirely comparable. However, further data is needed to move these speculative interpretations to being evidence-based. These preliminary differences may also suggest that POPAC has value by generating such emerging differences and study hypotheses for further exploration. Furthermore, the limited sample in this study compromised reliable analyses of differences between individual wards or type of staff due to questionable power. Further studies are needed to explore and explain the potential variance and/or confounding of ward and staff types.

POPAC ratings further indicate that staff in this sample report they often or very often assess the cognitive status of older patients upon admission, but that they rarely use evidence-based tools for these assessments. This finding is somewhat disconcerting given the multitude of available tools that exist for this purpose (e.g. the Mini–Mental State Examination, MMSE; the Short Portable Mental Status Questionnaire, SPMSQ; and the Confusion Assessment Method, CAM) and further evidence on the use of such tools in acute hospital processes would be valuable. A previous observational study at an acute care ward found that assessments of older patients’ cognition were based on the subjective judgments of staff rather than on evidence-based tools [1]. It seems reasonable to interpret this as being an inadequate assessment of older patients’ cognition that will contribute to less individualised care plans and less likelihood of PCC [1]. These findings also illustrate how POPAC can be used for practice development purposes, in addition to research. By highlighting processes that are interpreted as being less person-centred, management and staff can identify aspects of concern locally, as the POPAC content was based on internationally highlighted aspects of high quality care provision for this population. Thus, best practice interventions that target such concerns would seem highly relevant.

### Methodological considerations and ways forward

The study is limited by being based on a cross-sectional convenience sample of medically oriented inpatient wards. Further studies are needed in other settings and samples. The study is also limited by the 51% response rate, even though this can be considered within an acceptable range in academic studies [33]. The reasons for not responding are unclear and may have impacted on the results. These limitations need consideration when interpreting the results of this study.

The original three-factor dimensionality needs further exploration and more data is needed before conclusions can be made regarding dimensionality and reliability on subscale levels. Further testing of POPAC is also needed in terms of its applicability in research studies and practice development projects. Can the tool be used in person-centred interventions to explore the impact on practice as reported by staff, and connecting such estimates to perceived quality of care, workload, and costs? Further research in these areas would be desirable.

## Conclusions

The Swedish version POPAC is tentatively valid and reliable, and provides opportunities to measure perceived level of PCC for older patients with cognitive impairment in acute hospital services. It enables the accumulation of internationally comparable data for research and practice development purposes, and it can be used to explore the ideal and real care delivery processes for older patients with dementia and other cognitive impairments as reported by staff. POPAC can also be used in relation to different outcome variables to measure perceived impact of person-centred interventions on actual care practices and procedures as reported by staff. POPAC can highlight strengths as well as deficiencies in the perceived care of older patients, aspects that risk being overlooked in busy acute hospitals with a multitude of targets and deliverables. Detecting low values on specific items can help management and staff to make targeted interventions and clinical best practice improvements.

## Consent

Informed implied consent was obtained through the staff’ voluntary and anonymous return of the questionnaires.

## Competing interests

The authors declare they have no competing interests.

## Authors’ contribution

AN and DE conceived the study. AN carried out the data collection, analysed the data, interpreted the results and wrote the manuscript. BR, ML and DE participated in analyses, interpretation, and presentation, and drafted the manuscript. All authors read and approved the final manuscript.

## Pre-publication history

The pre-publication history for this paper can be accessed here:

http://www.biomedcentral.com/1472-6963/13/327/prepub
